# Deep Sequencing of Small RNAs in the Whitefly *Bemisia tabaci* Reveals Novel MicroRNAs Potentially Associated with Begomovirus Acquisition and Transmission

**DOI:** 10.3390/insects11090562

**Published:** 2020-08-23

**Authors:** Daniel K. Hasegawa, Md Shamimuzzaman, Wenbo Chen, Alvin M. Simmons, Zhangjun Fei, Kai-Shu Ling

**Affiliations:** 1 USDA, Agricultural Research Service, U.S. Vegetable Laboratory, 2700 Savannah Hwy, Charleston, SC 29414, USA; Daniel.Hasegawa@usda.gov (D.K.H.); Md.Shamimuzzaman@usda.gov (M.S.); Alvin.Simmons@usda.gov (A.M.S.); 2 Boyce Thompson Institute, 533 Tower Road, Ithaca, NY 14853, USA; chenwenbo@zju.edu.cn (W.C.); zf25@cornell.edu (Z.F.); 3 USDA, Agricultural Research Service, Robert W. Holley Center for Agriculture and Health, 538 Tower Road, Ithaca, NY 14853, USA

**Keywords:** whitefly, *Bemisia tabaci*, tomato yellow leaf curl virus, TYLCV, microRNA (miRNA)

## Abstract

**Summary:**

The whitefly (*Bemisia tabaci*), a notorious insect vector, transmits hundreds of viruses causing serious yield losses in a diverse food and fiber crops including beans, cassava, cotton, cucurbits, pepper, sweet potato and tomato, and results in billions of U.S. dollars of economic losses annually worldwide. To investigate the molecular mechanisms regulating gene expression in whitefly that is associated with begomovirus transmission, we conducted small RNA sequencing and compared the microRNA (miRNA) profiles between viruliferous whiteflies feeding on tomato plants infected with a begomovirus, tomato yellow leaf curl virus (TYLCV), and those whiteflies feeding on uninfected plants. We uncovered a comprehensive microRNA genetic regulatory system in whiteflies that may be involved in virus acquisition and transmission. Interestingly, correlating the expression profile of miRNAs and their target transcript expression in our earlier transcriptome study, we found miRNA expression was inversely correlated with predicted target gene expression in over 50% of all cases. This fundamental understanding will help identify new target sequences that could be used to improve RNA interference technology for whitefly control. These analyses could also serve as a model to study gene regulation in other systems involving arthropod transmission of viruses to plants and animals.

**Abstract:**

The whitefly *Bemisia tabaci* (Gennadius) is a notorious insect vector that transmits hundreds of plant viruses, affecting food and fiber crops worldwide, and results in the equivalent of billions of U.S. dollars in crop loss annually. To gain a better understanding of the mechanism in virus transmission, we conducted deep sequencing of small RNAs on the whitefly *B. tabaci* MEAM1 (Middle East-Asia Minor 1) that fed on tomato plants infected with tomato yellow leaf curl virus (TYLCV). Overall, 160 miRNAs were identified, 66 of which were conserved and 94 were *B. tabaci*-specific. Among the *B. tabaci*-specific miRNAs, 67 were newly described in the present study. Two miRNAs, with predicted targets encoding a nuclear receptor (*Bta05482*) and a very-long-chain (3R)-3-hydroxyacyl-CoA dehydratase 2 (*Bta10702*), respectively, were differentially expressed in whiteflies that fed on TYLCV-infected versus uninfected plants. To better understand the regulatory effects of identified miRNAs and their target genes, we correlated expression profiles of miRNAs and their target transcripts and found that, interestingly, miRNA expression was inversely correlated with the expression of ~50% of the predicted target genes. These analyses could serve as a model to study gene regulation in other systems involving arthropod transmission of viruses to plants and animals.

## 1. Introduction

The whitefly *Bemisia tabaci* (Gennadius) (Hemiptera: Aleyrodidae) is a globally-distributed insect pest that is capable of transmitting hundreds of pathogenic viruses to agricultural crops including tomato (*Solanum lycopersicum* L.), sweetpotato (*Ipomoea batatas* (L.) Lam.), beans (e.g., *Phaseolus* spp., melon (e.g., *Cucumis melo* L.), squash (*Cucurbita* spp.), and cassava (*Manihot esculenta*) [1]. *B. tabaci* is a complex of cryptic species with indistinguishable morphology, but exhibits diversity in their host preference, reproductive incompatibility, insecticide resistance, endosymbiont populations, and virus transmission [2,3,4]. Among the *B. tabaci* complex, two of the most widespread and damaging populations are *B. tabaci* MEAM1 (Middle East-Asia Minor 1) and *B. tabaci* MED (Mediterranean) [5,6]. Viral species belonging to five genera can be transmitted by *B. tabaci*, with the majority of species belonging to the *Begomovirus* genus (Family: *Geminiviridae*). Begomoviruses possess single-stranded, circular DNA genomes of ~2700 nt, and are exclusively transmitted by the whitefly *B. tabaci* in a persistent, circulative manner [7,8].

One of the most economically important begomoviruses transmitted by *B. tabaci* is tomato yellow leaf curl virus (TYLCV) [9], which has been the focus of numerous studies to better understand protein interactions between whiteflies and begomoviruses [10,11,12]. Transcriptome profiling has also been used to help understand the molecular mechanism of the whitefly–begomovirus relationship, including whitefly transcriptome responses to acquiring TYLCV [13,14,15] and tomato yellow leaf curl China virus (TYLCCNV) [16]. Although some basic information on the profiling of microRNAs (miRNAs) in the whitefly *B. tabaci* MEAM1 and MED are available [17,18,19], as is information on the role of miRNAs in other insect–virus relationships [20,21], there is need for additional knowledge regarding *B. tabaci* miRNAs that are related to virus acquisition, transmission, or feeding on a virus-infected plant.

MicroRNAs are endogenous non-coding small RNAs (19–25 nucleotides) that post-transcriptionally regulate gene expression in diverse organisms. Insect miRNAs are involved in regulation of various processes, including metabolism, development, apoptosis, and innate immune responses [22]. In *Drosophila melanogaster*, the miRNA pathway is initiated with transcription of a genome-encoded miRNA gene, which is processed in the nucleus by two RNAse III-like enzymes, Drosha and Pasha to generate pre-miRNAs (~70 nt) [22]. Pre-miRNAs are then exported into the cytoplasm and processed by Loquacious and another RNAse III enzyme, Dicer-1, into 21–23 nt duplexed RNAs. The miRNA duplex is loaded into an Argonaut-containing RNA-induced silencing complex (RISC) [22] and further processed to generate a RISC-loaded, single-stranded, mature miRNA that recognizes complementary messenger RNA (mRNA). Together, the successful pairing of RISC-loaded mature miRNAs with mRNAs silences the expression of protein-coding genes in both somatic and germ cells [23].

Here, we were interested in characterizing miRNA profiles in the whitefly *B. tabaci* MEAM1 when fed on a TYLCV-infected tomato plant. Using small RNA deep sequencing with three biological replicates per treatment, whitefly miRNA expression profiles were captured after feeding on either TYLCV-infected or uninfected tomato for 24 h, 48 h, and 72 h. We confirmed the presence of conserved miRNAs and identified a large number of novel *B. tabaci*-specific miRNAs. Interestingly, comparative expression analysis between miRNAs and their predicted mRNA targets from a previous transcriptome study [13] revealed a pattern of inverse relationship in 50% of the predicted genes.

## 2. Materials and Methods

### 2.1. Whiteflies and Feeding Assays

A full description of the methods on whitefly colony and feeding assays can be found in Hasegawa et al., 2018 [13]. Briefly, a whitefly *B. tabaci* MEAM1 colony was maintained at the U.S. Vegetable Laboratory on broccoli (*Brassica oleracea* L. var. *botrytis*) in a greenhouse (26 °C ± 5 °C). The MEAM1 colony was confirmed by PCR primers targeting the mitochondrial cytochrome oxidase 1 gene [24]. Approximately 1500 adult whiteflies were transferred to TYLCV-infected or uninfected tomato (*S. lycopersicum* cv. Moneymaker) plants and allowed to feed for 24, 48, or 72 h. Tomato cuttings were placed in a flask of water, sealed with parafilm, and placed inside of an insect-proof cage under controlled conditions set to 28 ± 1 °C, 14:10 (L:D) h photoperiod, and ~60% relative humidity. At the end of each time point, 200–500 living whiteflies were collected and immediately stored at −80 °C until processing. Three biological replicates were performed for each treatment. Tomato plants were verified for TYLCV-infection by PCR using primers to the coat protein (Table S5 and Hasegawa et al. [13]). The same pools of total RNA from whiteflies that fed on TYLCV-infected tomato and uninfected tomato used in our previous RNA-Seq study [13] were used in the current study. To determine the percent of whiteflies that had acquired TYLCV, PCR analysis was conducted using DNA preparations from each of 10 individual whiteflies per treatment feeding on TYLCV-infected plants for each acquisition access period (AAP). The results showed that 60% (6 of 10) of whiteflies were positive for TYLCV at 24 h, and 100% (10/10) were positive for either 48 h or 72 h [13]. As expected, individual whiteflies that were exposed to uninfected tomato plants tested negative for TYLCV.

### 2.2. RNA Isolation and Library Preparation

Total RNA was isolated with TRIzol Reagent (Thermo Fisher Scientific, Waltham, MA, USA) according to the manufacturer’s protocol and purified with the Direct-zol RNA MiniPrep kit (Zymo Research, Irvine, CA, USA). Small RNAs were then PAGE-purified and libraries were prepared, as previously described [25], and sequenced on an Illumina HiSeq 2000.

### 2.3. Initial Sequence Processing and Analysis of Reads for 18 Small RNA Libraries

Small RNA sequence reads from all 18 libraries were processed to remove adapters and low-quality reads using a Perl script provided in the VirusDetect package [26]. Sequences that were shorter than 15 nt and longer than 40 nt were removed. The high quality and size of the selected reads were then aligned to the Rfam database (Version 12.1) (http://rfam.sanger.ac.uk/) to filter out reads of rRNAs, tRNAs, and snRNAs, and the remaining cleaned reads were used for miRNA identification.

### 2.4. Identification of Conserved and Novel miRNAs

The small RNA reads (19–25 nt) were aligned to the insect miRNA sequences (*Drosophila melanogaster, Aedes aegypti, Apis mellifera, Tribolium castaneum, and Bombyx mori*) that were available in miRBase (v21 http://www.miRBase.org/) [27] to identify conserved miRNAs. Specifically, BLASTN searches were performed against insect mature miRNA sequences with an E-value cut-off value of 10. Alignments that had less than or equal to four nucleotide mismatches were defined as conserved miRNAs. Novel miRNA candidates were identified using miRDeep2 [28] with default parameters by mapping small RNA reads to the whitefly genome [29] and predicting pre-miRNA precursor hairpin structures. Secondary structures for all predicted pre-miRNAs as well as detailed summaries for each miRNA were obtained, including information about the Dicer cleavage site, minimum folding free energy, and the number of reads. Absolute read numbers were normalized as reads per million (RPM) prior to comparative analysis.

### 2.5. Validation of Novel miRNAs by Reverse Transcription Polymerase Chain Reaction (RT-PCR)

Small RNAs from the 48 h time point (uninfected treatment, first biological replicate) were purified from a 15% urea-TBE-polyacrylamide gel as previously described [25], and selected miRNAs were specifically detected using the Poly(T) Adaptor RT-PCR protocol [30]. Small RNAs were first poly(A) tailed using the *E. coli* Poly(A) Polymerase (New England Biolabs, Ipswich, MA, USA) and then reverse transcribed using the SuperScript III First-Strand Synthesis for RT-PCR kit (Thermo Fisher Scientific, Waltham, MA, USA) and a poly(T) adaptor primer [30] (Table S5). PCR reactions were assembled in 20 μL triplicate reactions using 100 ng cDNA, miRNA-specific forward primer, poly(T) adaptor reverse primer (Table S5), and 2X Brilliant II SYBR Green QPCR Low ROX Master Mix (Agilent Technologies, Santa Clara, CA, USA). Reactions were performed using a Stratagene Mx3000p system (Stratagene, San Diego, CA, USA), and Ct values and dissociation curves were analyzed for specific amplification. Non-template control reactions were also simultaneously set up in triplicate, none of which yielded a Ct value or a dissociation curve (data not shown). PCR products were then analyzed on an agarose gel (4%) to verify the size of the amplimer.

### 2.6. Target Prediction and Gene Ontology Analysis

MicroRNA targets were predicted using a published whitefly dataset [29] and a widely used miRNA target predictor, miRanda (v3.3, http://www.microrna.org/microrna/getDownloads.do) [18]. Alignment scores greater than or equal to 150 and miRNA/mRNA binding energy (Minimum Free Energy (MFE, ΔG)) less than −20 kcal/mol were used to run the program. The miRanda output revealed many targets for individual miRNA. Predicted targets were further filtered using more stringent criteria (alignment score greater than or equal to 170, minimum free energy (MFE) less than −25 kcal/mol, minimum alignment length greater than or equal 15 nt) to obtain the most probable targets. The predicted target for each miRNA that had the highest alignment score was kept and compiled into a list of predicted single targets for all miRNAs. Gene ontology (GO) term analysis was performed on predicted single miRNA targets using Blast2GO [31].

## 3. Results

### 3.1. Overview of miRNA Data

To capture whitefly miRNA profiles in response to feeding on TYLCV-infected tomato, deep sequencing of small RNAs was performed after whiteflies fed on either TYLCV-infected tomato or uninfected tomato for acquisition access periods (AAP) of 24 h, 48 h, and 72 h. Three biological replicates were performed per AAP and treatment. Overall, ~175 million reads were obtained from 18 libraries, with ~5–13 million reads per library. After adapter trimming and removing low quality reads together with filtering out reads mapped to rRNAs, tRNAs, and snRNAs, ~2–8 million cleaned reads with lengths of 15–40 nt were generated per library, with an average of ~71% of the reads mapped to the *B. tabaci* MEAM1 genome (http://www.whiteflygenomics.org [29]; Table S1). Pearson’s correlation coefficients were close to 1 for all biological replicates (Table S2), suggesting the data were highly reproducible.

**Figure 1:**
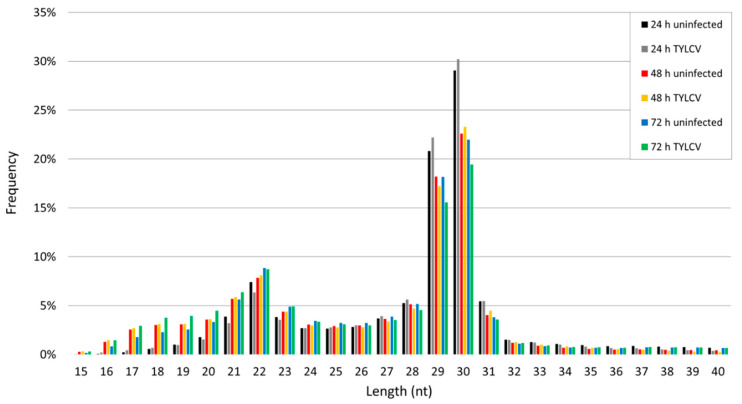
Frequency of read lengths. Distribution of small RNAs identified in whiteflies (*B. tabaci*) that fed on tomato yellow leaf curl virus (TYLCV)-infected tomato or uninfected tomato for 24, 48, and 72 h.

Small RNAs of 19–25 nt are likely to be made up of miRNAs, with the majority of miRNAs ranging from 21–23 nt. Overall, small RNAs ranging from 19–25 nt accounted for ~28% of all reads, while those ranging from 21–23 nt accounted for ~17% of all reads (Figure 1). In whiteflies that fed on uninfected or TYLCV-infected tomato, reads within the range of 21–23 nt in length accounted for 15% and 13% of all reads at 24 h, 18% and 18% of reads at 48 h, and 19% and 20% of reads at 72 h, respectively (Figure 1). Small RNAs of 26–31 nt in length are considered piRNAs and were the focus of a separate study reported in Shamimuzzaman et al. [32].

After aligning the selected small RNA reads (19–25 nt) to the whitefly *B. tabaci* MEAM1 genome [29], conserved and novel miRNAs were identified using miRDeep2 [28]. Overall, 160 miRNAs were identified, of which 66 were conserved and 94 were *B. tabaci-*specific (Table 1). A total of 54 conserved miRNA families were represented and 67 out of the 94 *B. tabaci-*specific miRNAs are newly described. When all 160 miRNAs were mapped to the *B. tabaci* MED genome [33], 50 out of 54 conserved miRNA families and 88 out of 94 *B. tabaci*-specific miRNAs were common between *B. tabaci* MEAM1 and MED, suggesting a high degree of conservation in different *B. tabaci* biotypes (Table S3).

### 3.2. Identification and Validation of Novel B. tabaci-Specific miRNAs

Among the 67 novel *B. tabaci*-specific miRNAs discovered in this study, a total of 17 had RPM (transcripts per million) values greater than 10 in at least one treatment at one time point (Table S4). The top five highly expressed novel miRNAs had RPM values greater than 200, miRDeep2 scores greater than 5000, and minimum free energy values less than −19 kcal/mol, with Bta-miRn88 as the most abundant novel miRNA, which was expressed at over 2000 RPM at 72 h (miRDeep2 score = 75,255; minimum free energy = −27 kcal/mol) (Table S4).

Using the whitefly *B. tabaci* MEAM1 genome sequence as a reference, we were able to predict pre-miRNA secondary structures for novel miRNAs identified in this study. Among them, seven pre-miRNA secondary hairpin structures are presented (Figure S1). These novel miRNAs were selected based on their expression values: top four highest expressed miRNAa, one lowest expressed miRNA, and two mid-range expressing miRNAs. To validate the miRNAs identified in the current study, six *B. tabaci*-specific miRNAs and two conserved miRNAs with various levels of expression (from low to high) were chosen. Three newly discovered miRNAs in this study (Bta-miRn23, Bta-miRn61, and Bta-miRn88 in Table 2), three known *B. tabaci*-specific miRNAs (Bta-miRn46, Bta-miRn47, and Bta-miRn99 in Table S6), and two conserved miRNAs (miR-307 and miR-316 in Table S7) were selected for validation using poly(T) adaptor real-time RT-PCR [30]. Briefly, small RNAs were poly(A)-tailed and then reverse transcribed using a poly(T) adaptor primer, and PCR was performed using a miRNA-specific forward primer and poly(T) adaptor reverse primer (See methods and Table S5). All eight reverse-transcribed miRNAs generated a single Ct value with a uniform dissociation curve, and when PCR products were visualized on a 4% agarose gel using electrophoresis, predicted products of reverse transcribed miRNAs with ligated adaptor sequences migrated to the expected location of 63–67 nt (Figure S2).

### 3.3. Predicted Targets for Novel and Conserved B. tabaci miRNAs

Predicting miRNA targets can be challenging because a single miRNA can have multiple targets and it can possess multiple functions, depending on the experimental conditions. We predicted targets for the identified miRNAs using miRanda [34]. All of the predicted target genes for each miRNA identified in this study are listed in Table S6. To obtain a high confidence miRNA target for each miRNA, we applied stringent filtering criteria. Candidate target genes had to possess an alignment score greater than or equal to 170, with a minimum free energy (MFE) less than −25 kcal/mol and a minimum alignment length greater than or equal to 15 nt. Based on these criteria, novel *B. tabaci-*specific miRNAs and their single target genes are presented in Table 2. Among the 17 highly expressed novel miRNAs (Table S4), several were predicted to target receptors, transcription factors, zinc finger proteins, centrosomal proteins, and proteins with unknown functions (Table 2). Among the 48 conserved miRNAs that had RPM values greater than 10, several were predicted to target receptors, transporters, transcription factors, and Cytochrome P450 (Table S7).

### 3.4. Differentially Expressed miRNAs with a Potential Association with TYLCV Transmission

Further analysis revealed that only two miRNAs were differentially expressed in whiteflies that fed on TYLCV-infected tomato compared to whiteflies fed on uninfected tomato. The first miRNA, Bta-miRn23, was downregulated in whiteflies fed on TYLCV-infected tomato at 48 h (fold change = −3.13; *p* = 0.01) and its single predicted target was Bta05482, which encodes for a nuclear receptor. The second miRNA, miR-1-3p, was downregulated at 72 h in whiteflies fed on TYLCV-infected tomato (fold change = −1.52; *p* = 0.03) and its single predicted target was Bta10702, which encodes the very-long-chain (3R)-3-hydroxyacyl-CoA dehydratase 2 (Table 3). Although there were three additional miRNAs (miR-996, miR-219, and miR-iab-4) that had fold change values greater than 1.5, their differential expression was not statistically significant (Table 3)

### 3.5. Evaluating Potential Inverse Relationships Between miRNA and mRNA Expression under the Same Treatment

Since miRNAs identified in the present study originated from the same pool of RNA preparations that were used to generate whitefly gene expression profile data [13], we used these data sets to explore the relationships between miRNA expression and mRNA expression. For 13 novel miRNAs that had predicted targets and RPM values > 10, ~50% of the miRNAs had inverse expression profiles with their predicted target transcripts (Table 2). For example, Bta-miRn88 had a fold change value of −1.16, while the predicted target gene Bta07821 had a fold change value of 1.02 in whiteflies fed on TYLCV-infected tomato compared to whiteflies that fed on uninfected tomato (Table 2). Specifically, 6, 8, and 4 miRNAs (out of a total of 13 miRNAs) had inverse expression values with their predicted targets at 24 h, 48 h, and 72 h (Table 2). The same trend was observed for all 160 miRNAs, such that ~50% of the miRNAs had inverse expression profiles with their predicted target transcripts (Table S8).

Considering miRNA target prediction can be challenging, we then looked at the top five predicted target genes for each of the miRNAs that had a fold change value > 1.5 (Table S9). The miRNA miR-996 had a fold change value of −1.72 (*p* > 0.05) at 24 h and 3 out of 5 predicted target genes had inverse expression values. For miR-219 (FC = −1.50, *p* > 0.05 at 24 h), 2 out of 5 predicted target genes had inverse expression values. For Bta-miRn23, which was significantly differentially expressed (FC = −3.13, *p* = 0.01 at 48 h), 2 out of 5 predicted target genes were inversely expressed. For miR-1-3p, which was also significantly differentially expressed (FC = −1.52, *p* = 0.03 at 72 h), 4 out of 5 target genes had inverse expression profiles (Table S9).

### 3.6. Gene Ontology Assignments for Predicted miRNA Targets

**Figure 2:**
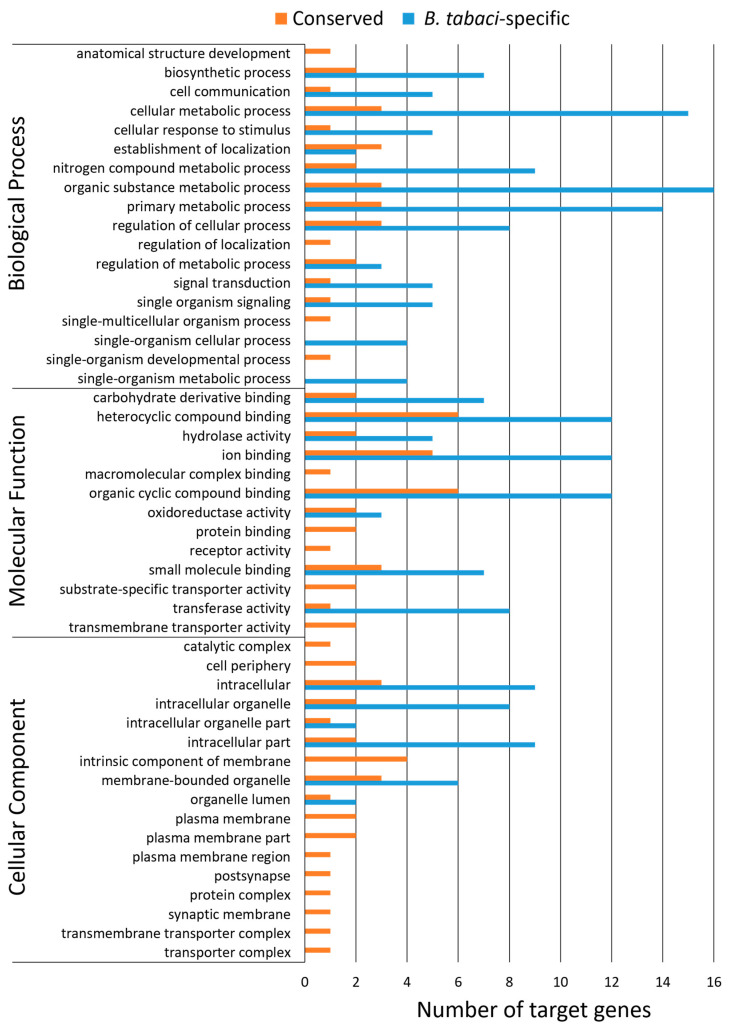
Overview of gene ontology for single gene targets of miRNAs. Functional classification of all 160 miRNAs. Number of conserved (orange) and *B. tabaci*-specific (blue) miRNAs assigned to each ontology. Gene ontology terms of level 2 are shown.

The predicted single gene targets for all 66 conserved miRNAs and 94 *B. tabaci-*specific miRNAs were assigned gene ontology (GO) terms using Blast2GO (Figure 2). Among the GO terms that were most represented were organic substance metabolic process (19 target genes from 16 *B. tabaci-*specific miRNAs and three conserved miRNAs), cellular metabolic process (18 target genes from 15 *B. tabaci-*specific miRNAs and three conserved miRNAs), organic cyclic compound binding and hetero-/organic cyclic compound binding (each with 18 target genes from 12 *B. tabaci-*specific miRNAs and six conserved miRNAs), and intracellular (12 target genes from nine *B. tabaci-*specific miRNAs and three conserved miRNAs) (Figure 2). Two GO terms, single-organism cellular and single-organism metabolic processes, were represented by only *B. tabaci-*specific miRNAs (Figure 2). The four miRNAs representing this category were Bta-miRn89, Bta-miRn40, Bta-miRn75, and Bta-miRn74, and had respective target genes, *Bta12365* encoding an elongation of very long chain fatty acids protein, *Bta00047* encoding a type II inositol-1,4,5-trisphosphate 5-phosphatase, *Bta08249* encoding a thioredoxin M, and *Bta08132* encoding a ribose-phosphate pyrophosphokinase 1.

## 4. Discussion

Tomato yellow leaf curl virus and other begomoviruses are exclusively transmitted by the *B. tabaci* in a persistent-circulative manner, such that once ingested by an adult whitefly, virions pass to the midgut, where they move across the midgut membrane to the hemolymph [5,35], and circulate back into the primary salivary glands, where they are egested with saliva during insect feeding [7,36,37]. By analyzing miRNA expression in the present study, and in reference to the relevant mRNA expression from our previous study [13], we gained insight into global gene expression and regulation of whitefly genes potentially associated with TYLCV transmission.

This report identified a total of 160 miRNAs in the whitefly *B. tabaci* MEAM1. When all 160 miRNAs were mapped to the *B. tabaci* MED genome [33], a high level of conservation between MEAM1 and MED was observed (93% of conserved miRNA families in *B. tabaci* MEAM1 mapped to MED and 96% of *B. tabaci* MEAM1-specific miRNAs mapped to MED). However, a few miRNAs were unique to *B. tabaci* MEAM1, suggesting possible differences in the miRNA regulatory mechanisms.

Among the 160 miRNAs, 66 were conserved among insects and 94 were specific to *B. tabaci* MEAM1. Further analysis revealed that the conserved miRNAs fell into 54 miRNA families and 67 of the 94 *B. tabaci-*specific miRNAs were newly identified in this study. The current study identified fewer conserved miRNAs than previous reports [17,18,19], which is likely due to differences in the parameters used. Specifically, conserved miRNAs were limited to the insect database in miRBase (http://www.miRBase.org/), which could have had a dramatic impact on the number of miRNAs identified. For example, analysis of the highly conserved *let-7* family of miRNAs revealed that only a single isoform was identified from our study (*let-7-5p*), whereas up to eight isoforms (*let-7, -7a-5p, -7b-5p, -7c-5p, -7d-5p, -7e-5p, -7f-5p, -7g*) were identified in other studies [17,18]. Although the *let-7* family is highly conserved, there is variation in the number of isoforms among animal species [38]. For example, only a single isoform of *let-7* has been described in the nematode (*Caenorhabditis elegans*) and fruit fly (*D. melanogaster*) [38], and a manual search in miRBase (v.22, http://www.miRBase.org/) revealed that the presence of a single *let-7* isoform extended to other insects including mosquito (*A. aegypti*), red flour beetle (*T. castaneum*), silkworm (*B. mori*), and pea aphid (*Acyrthosiphon pisum*). In contrast, higher animals such as fish and mammals have diverse *let-7* family members, including *let-7a, -7b, -7c, -7d, -7e, -7f, -7g, -7h, -7i, -7j, -7k* [38], suggesting that additional *let-7* isoforms identified in the previous studies may have been mapped to animals other than insects. This suggests that differences in the parameters used to identify conserved miRNAs in whiteflies may have contributed to the large differences in the total number of conserved miRNAs and total number of conserved miRNA families described in the current study.

In this study, small RNA libraries were prepared from the same pool of total RNAs isolated from whiteflies feeding on tomato plants either infected with TYLCV or non-infected controls, in which differential expression of mRNAs were evaluated using transcriptome analysis [13]. Therefore, we sought to characterize miRNA expression patterns with respect to the predicted target transcript expression. Surprisingly, only two miRNAs were differentially expressed (*p* < 0.05) and only five miRNAs had fold changes greater than 1.5 (*p* > 0.05) in whiteflies that fed on TYLCV-infected tomato versus uninfected tomato.

One possibility for observing very few changes in miRNA expression is that the expression of miRNAs occurs in a tissue-specific manner, as suggested in other insects [38] and, therefore, miRNAs were not captured during the sequencing of small RNAs isolated from whole-body whiteflies. Another possibility might have to do with the timing of virus acquisition and circulation in the whitefly body. Considering that whiteflies were allowed to feed on TYLCV-infected plants for different periods of time, virion ingestion and circulation could have varied between individual whiteflies, thus activating miRNA expression at different time points. This is supported by our finding that TYLCV acquisition was relatively slow, such that only 60% of the whiteflies tested positive for TYLCV at 24 h, while 100% of whiteflies tested positive for the virus at 48 h and 72 h post feeding [13]. Furthermore, when the miRNA expression data was referenced to the gene expression data, none of the predicted miRNA targets matched the genes that were differentially expressed in the transcriptome, suggesting that miRNA target prediction may be challenging. This is likely due to the nature of miRNA sequences, such that mismatches can occur between miRNAs and their targets, and a single miRNA can control many transcripts [39,40]. Therefore, further studies would be necessary to validate the targets experimentally.

## 5. Conclusions

Together with our earlier report on the analysis of differential gene expression in whiteflies fed on TYLCV-infected and uninfected tomato plants [13], in combination with the genome-wide profiling of piRNAs [32] and the miRNAs analysis in the present study, we have achieved a comprehensive insight into the possible genes and regulatory elements that might be involved or associated with acquisition, circulation, and transmission of a begomovirus, specifically TYLCV in the whitefly, *B. tabaci*. Overall, this study reports a suite of newly described whitefly *B. tabaci*-specific miRNAs and contributes to the growing knowledge of whitefly biology in association with begomovirus transmission. The identified differentially expressed miRNAs may serve as potential targets in designing new RNAi constructs to interfere with virus transmission by whiteflies.

## Figures and Tables

**Table 1:** Number of conserved and *B. tabaci*-specific microRNAs identified in this study.

Length	No. miRNAs		
	Conserved	*B. tabaci*-Specific	Total
19	1	4	5
20	0	1	1
21	22	9	31
22	32	61	93
23	10	14	24
24	1	2	3
25	0	3	3
Total	66	94	160

**Table 2:** Fold change values for novel miRNAs and their predicted single target genes in whiteflies (*B. tabaci*) fed on TYLCV-infected tomato.

Name	Target	Annotation	24 h		48 h		72 h	
			miRNA	Target Gene *	miRNA	Target Gene *	miRNA	Target Gene *
Bta-miRn88	Bta07821	Zinc finger CCCH domain-containing protein 14	−1.16	1.02	−1.03	1.27	1.03	1.01
Bta-miRn61	Bta03447	Cadherin EGF LAG seven-pass G-type receptor 3	−1.20	1.07	1.10	−1.04	−1.10	−1.09
Bta-miRn100	Bta13203	FGGY family of carbohydrate kinase	−1.21	−1.25	1.01	−1.20	−1.03	−1.05
Bta-miRn62	Bta09225	UPF0472 protein C16orf72	−1.30	1.11	−1.02	1.05	1.17	1.19
Bta-miRn54	Bta07082	Centrosomal protein of 78 kDa	−1.05	−1.39	1.17	1.19	1.44	1.06
Bta-miRn108	Bta00358	Dynamin-1-like protein	−1.16	−1.04	−1.03	1.05	1.10	1.12
Bta-miRn71	Bta05170	Unknown protein	1.00	12.00	1.01	−1.38	1.17	−1.30
Bta-miRn69	Bta13835	Unknown protein	−1.19	1.10	−1.03	−1.01	1.02	1.31
Bta-miRn72	Bta08675	Unknown protein	−1.27	−1.38	1.07	1.12	−1.11	−1.06
Bta-miRn112	Bta04487	Transcription factor BTF3-like protein 4	1.23	−1.08	−1.15	−1.06	1.05	−1.12
Bta-miRn58	Bta04052	Centrosomal protein of 164 kDa	−1.21	−1.04	−1.03	1.19	1.03	−1.06
Bta-miRn93	Bta09366	Zinc finger protein 250	−1.14	1.12	1.25	−1.19	−1.13	1.12
Bta-miRn23	Bta05482	Nuclear receptor	−1.44	−1.09	−3.13	−1.23	−1.04	−1.73

* Fold change values for novel miRNAs and their predicted single target genes in whiteflies (*B. tabaci*) fed on TYLCV-infected tomato. Fold change values for target genes were obtained from the dataset of Hasegawa et al. (2018). Both miRNA and mRNA were isolated from the same pool of total RNA. Blue indicates downregulation of the miRNA/target gene in whiteflies fed on TYLCV-infected tomatoes compared to whiteflies that fed on uninfected tomatoes, while red indicates upregulation. Color intensities (from blue to red) reflect relative expression levels.

**Table 3:** MicroRNAs with fold change > 1.5 and reads per million (RPM) > 10 in whiteflies (*B. tabaci*) fed on TYLCV-infected tomato vs. uninfected tomato.

**Name**	**Sequence**	**Length**	**Target**	**Annotation**	**Fold Change**		
					**24 h**	**48 h**	**72 h**
miR-996	ugacuagaguuacacucguca	21	Bta08371	Zinc finger protein, putative	−1.72	1.05	1.37
miR-219	ugauuguccaaacgcaauucuug	23	Bta10860	Beta-1,3-galactosyltransferase, putative	−1.50	−1.00	1.26
Bta-miRn23	ccccucgccgcgcggagcu	19	Bta05482	Nuclear receptor	−1.44	−3.13 *	−1.04
miR-1-3p	uggaauguaaagaaguauggag	22	Bta10702	Very-long-chain (3R)-3-hydroxyacyl-CoA dehydratase 2	−1.20	1.25	−1.52 *
miR-iab-4	acguauacuaaauguauccuga	22	None	N/A	−1.27	1.05	1.53

* asterisks indicate a significant change of *p* < 0.05.

## Data Availability

Small RNA sequence reads have been deposited in the NCBI GEO as accession GSE111343.

## Supplementary Materials

The following are available online at https://www.mdpi.com/2075-4450/11/9/562/s1, Figure S1: Predicted secondary structures for seven *B. tabaci*-specific miRNAs with various hairpins, Figure S2: RT-PCR validation of *B. tabaci*-specific and conserved miRNAs, Table S1: Summary of reads after trimming, filtering, and mapping to the whitefly genome, Table S2: Pearson’s correlation coefficients for three biological replicates, Table S3: microRNAs found in *B. tabaci* MEAM1 and MED, Table S4: Novel miRNAs identified from this study with RPM > 10, Table S5: Primers used in this study, Table S6: All predicted targets for miRNAs identified in the current study, Table S7: Conserved miRNAs identified from this study with RPM > 10, Table S8: Fold change values for all 160 miRNAs and their predicted target genes in whiteflies fed on TYLCV-infected tomato, Table S9: Fold change values for miRNAs (fold change > 1.5 and RPM > 10) and their top five predicted target genes in whiteflies fed on TYLCV-infected tomato.
