# Supplementary material for: Deep Sequencing of Small RNAs in the Whitefly Bemisia tabaci Reveals Novel MicroRNAs Potentially Associated with Begomovirus Acquisition and Transmission
Source: Insects. 2020 Aug 23;11(9):562. doi: 10.3390/insects11090562 (PMC7564577; doi:10.3390/insects11090562)

Supplementary Figure S1: Predicted secondary structures for seven *B. tabaci*-specific miRNAs with various hairpins. Secondary miRNA structures were predicted using miRDeep2. Red = mature region; yellow = stem loop; purple/blue = star region.

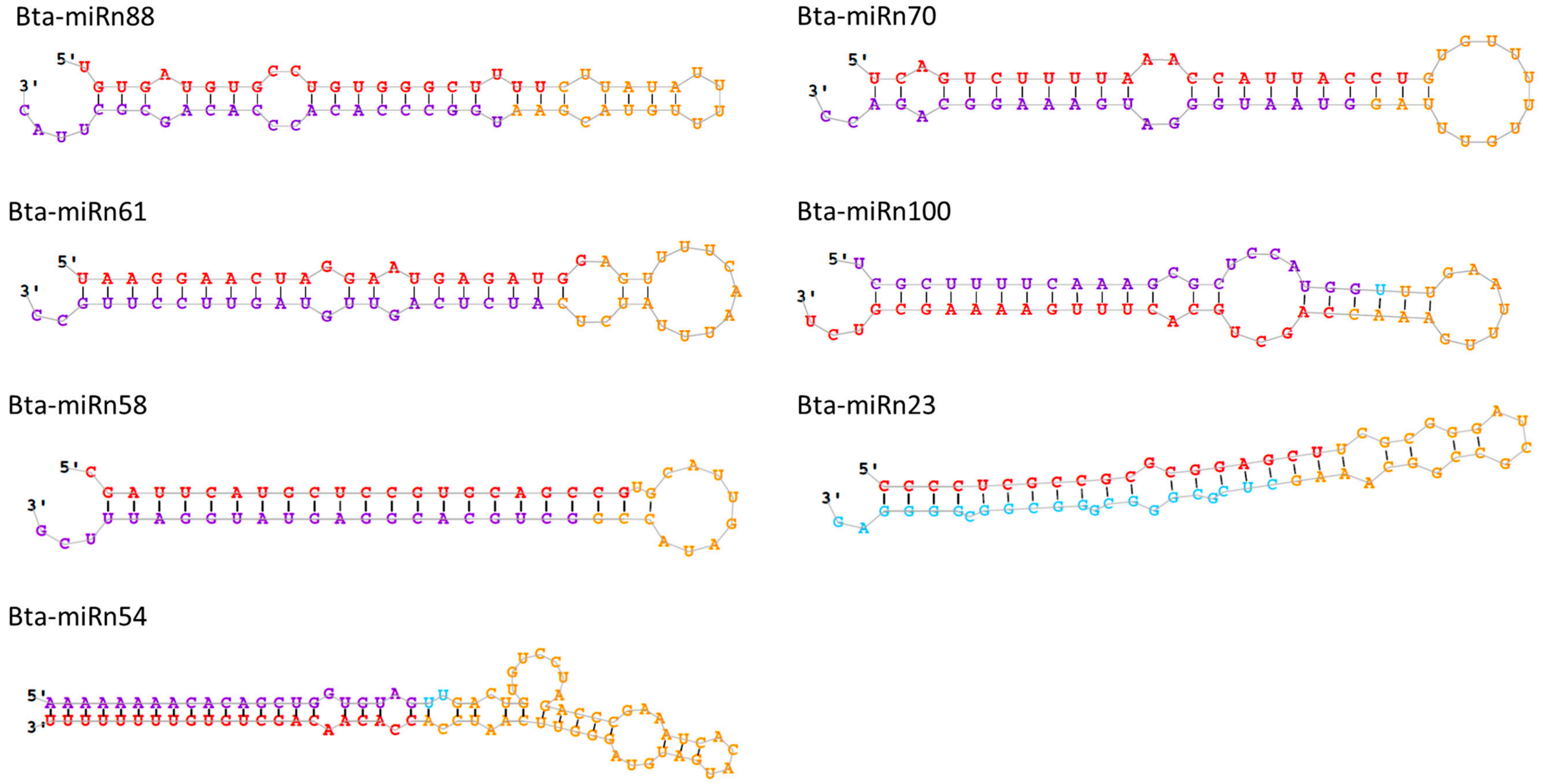

Supplementary Figure S2: RT-PCR validation of *B. tabaci*-specific and conserved miRNAs. Poly(T) Adaptor RT-PCR was used to validate six *B. tabaci*-specific miRNAs and two conserved miRNAs. A) Plots for each miRNA are shown to illustrate Ct values and dissociation curves. B) The resulting PCR products were ran on an agarose gel.

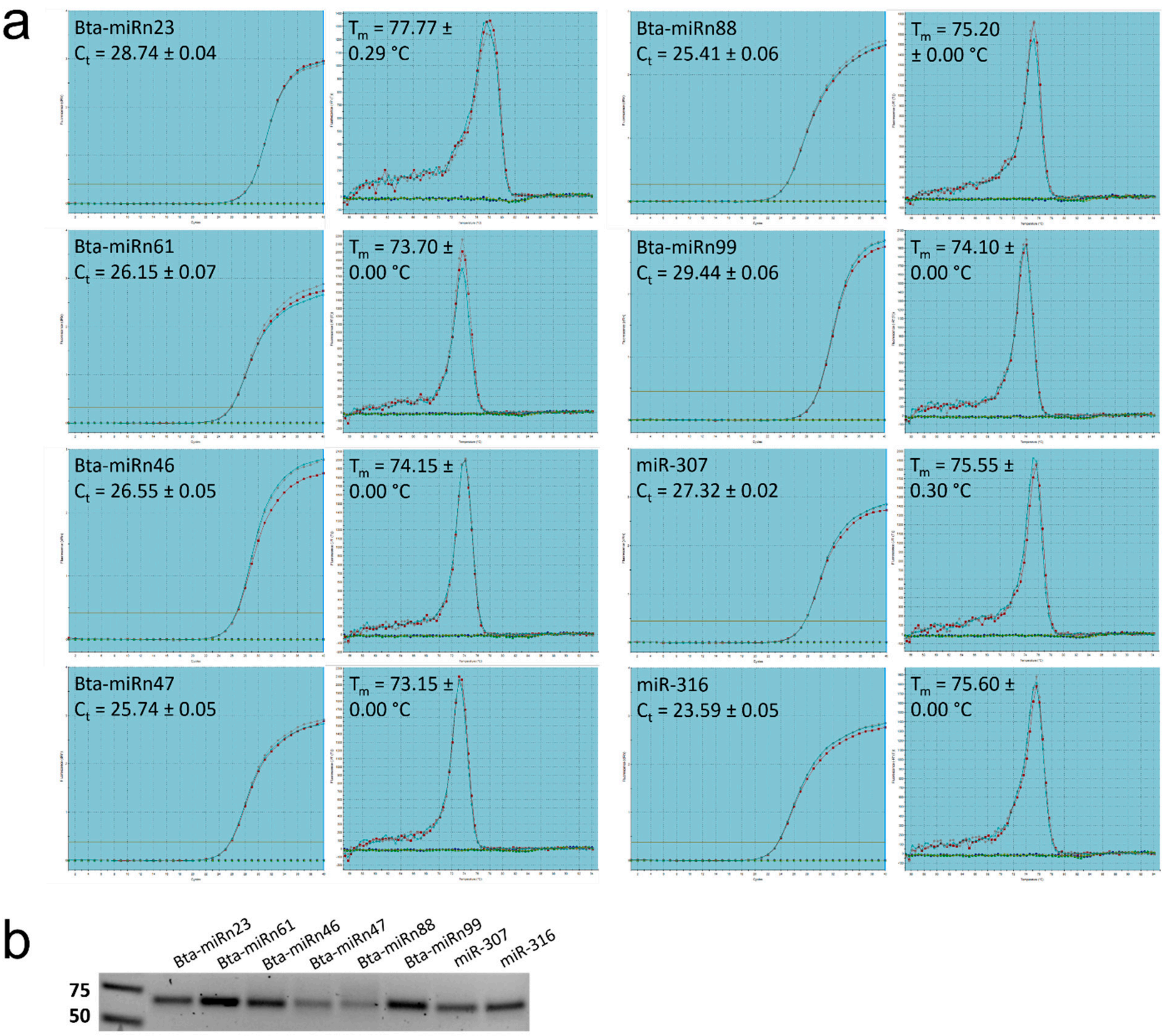

Supplement: Supplementary file 1 [file insects-11-00562-s001.zip › insects-870662 - suppl figures.pdf]
